# Are changes in olanzapine-induced liver enzyme levels associated with *GSTT1*, *GSTM1*, *GSTP1*, and *OGG1* gene polymorphisms?

**DOI:** 10.2478/aiht-2024-75-3770

**Published:** 2024-03-29

**Authors:** Aylin Elkama, Nazlıcan İlik, Mehmet Ak, Bensu Karahalil

**Affiliations:** Gazi University Faculty of Pharmacy, Department of Toxicology, Ankara, Turkey; Necmettin Erbakan University, Meram Faculty of Medicine, Department of Psychiatry, Konya, Turkey; Eastern Mediterranean University Faculty of Pharmacy, Department of Toxicology, Famagusta, North Cyprus

**Keywords:** α-GST, ALT, AST, drug-induced liver injury, mental disorders, psychotropic drugs, α-GST, ALT, AST, mentalni poremećaji, lijekom prouzročeno oštećenje jetre, psihotropni lijekovi

## Abstract

Olanzapine treatment sometimes produces transient liver biochemistry abnormalities, and such drug-induced liver injuries are mainly monitored by measuring blood levels of alanine aminotransferase (ALT) and aspartate aminotransferase (AST), whereas alpha-glutathione-S-transferase (α-GST) is not routinely measured in clinics, even though it can serve as an earlier and more specific biomarker of liver damage. Susceptibility to drug-induced liver injury can much depend on the gene polymorphisms regulating the activity of DNA detoxification and repair enzymes. The aim of this study was to evaluate which of the three liver enzymes – α-GST, ALT, and AST – is the most sensitive biomarker of olanzapine-induced liver injury and how their blood levels are affected by the *GSTT1*, *GSTM1*, *GSTP1,* and *OGG1* gene polymorphisms in 30 olanzapine-treated patients. Contrary to our hypothesis, the increase in serum α-GST levels was not significantly greater than that of the transaminases. ALT turned out to be an earlier biomarker of liver injury than the other two enzymes. No significant association was found between gene polymorphisms and liver enzyme levels, save for *GSTP1 Ile/Val + Val/Val* and ALT, which points to this genotype as a risk factor for drug-induced liver injury. Future studies might help to identify the underlying mechanisms of transient liver enzyme increase associated with this genotype.

## Abstract (hr)

Liječenje olanzapinom može dovesti do prolaznih abnormalnosti u jetrenim biokemijskim nalazima i takva se lijekom prouzročena oštećenja jetre obično nadziru mjerenjem razina alanin aminotransferaze (ALT) i aspartat aminotransferaze (AST) u krvi, a alfa-glutation-S-transferaza (α-GST) ne mjeri se rutinski u klinici premda može poslužiti kao raniji i specifičniji biomarker oštećenja jetre. Osjetljivost jetre na lijekove može dosta ovisiti o polimorfizmu gena regulatora aktivnosti enzima uključenih u procese detoksifikacije i popravka DNA. Cilj je ovoga istraživanja bio ocijeniti koji je od triju jetrenih enzima – α-GST, ALT i AST – najosjetljiviji biomarker olanzapinom prouzročenog oštećenja jetre te kako polimorfizmi gena *GSTT1*, *GSTM1*, *GSTP1* i *OGG1* utječu na njihove razine u 30 bolesnika koji su primali taj lijek. Suprotno našoj hipotezi, povišenje serumskih razina α-GST-a nije bilo značajno više od povišenja razina transaminaza. ALT se pokazao ranijim biomarkerom oštećenja jetre od ostalih dvaju enzima. Osim toga, nismo otkrili značajnu povezanost između genskih polimorfizama i razina enzima, izuzev za povezanost genotipa *GSTP1 Ile/Val + Val/Val* s ALT-om, što upozorava na povećani rizik od lijekom prouzročenog oštećenja jetre u nositelja ovoga genotipa. Buduća bi istraživanja mogla pomoći u prepoznavanju mehanizama uslijed kojih dolazi do prolaznog povišenja razina ovog jetrenog enzima povezanoga s ovim genotipom.

Olanzapine has been approved for use in patients with schizophrenia, bipolar I disorder, and resistant depression. Long-term treatment can increase the levels of liver enzymes in up to 50 % of patients and impair the liver function, leading to clinically visible hepatitis with jaundice. The time to the onset of liver injury varies between a few weeks and a year (1). It is therefore important to detect adverse drug reaction early, especially in genetically susceptible individuals. Liver function in patients receiving psychotropic drugs such as olanzapine is usually monitored through blood liver enzymes, namely aspartate aminotransferase (AST) and alanine aminotransferase (ALT) (2). Some authors claim that an earlier and even more specific marker of liver injury is alpha-glutathione-S-transferase (α-GST), since it is highly concentrated in liver cell cytosol, widely distributed throughout the liver, and rapidly released from damaged hepatocytes into the plasma in large quantities, yet has a short half-life in plasma (3, 4). Furthermore, α-GST is not elevated in cases of muscle damage, haemolysis, or extrahepatic inflammation such as rheumatoid arthritis, and is therefore more liver-specific than aminotransferases (5). Even so, it is not routinely tested in clinics.

Recent research (6, 7) has established an association between genetic polymorphisms regulating GST (and other drug-metabolising) enzymes and the incidence of drug-induced liver injury (6, 7), as the GST superfamily plays a vital role in reducing oxidative liver cell injury and in the efficacy of drug treatment (8, 9). Cytosolic GSTs are divided into eight sub-classes and are highly polymorphic. Gene deletions caused by homozygous null mutation of *GSTM1* and *GSTT1*, for instance, have been reported to significantly increase the risk of liver injury (9, 10). In Caucasians *GSTT1* and *GSTM1* null genotypes are found in 20 % and 38–67 % of the population, respectively (8). Furthermore, the levels of GSTM1 are significantly lower in patients with major depression and schizophrenia compared to healthy controls, which suggests that these patients are more prone to oxidative damage (11). In addition, the *G* allele in the *GSTP1 A313G* polymorphism is associated with non-alcoholic fatty liver disease and antituberculosis drug-induced liver injury (8, 12).

Another important enzyme involved in DNA repair is 8-oxoguanine DNA glycosylase 1 (OGG1). It is responsible for the base excision of 7,8-dihydro-8-oxoguanine (8-OHG or 8-OHdG), the most critical DNA lesion resulting from oxidative stress caused by ROS. Its repair ability, however, may depend on the *OGG1* gene polymorphisms. For example, the homozygous *OGG1 Ser326Cys* polymorphism has been associated with lower DNA repair activity and increased risk of oxidative stress-related diseases, including cancer (13).

There are several studies associating schizophrenia with *GST* (14,15,16) or *OGG1* genotypes (17), but none have established higher susceptibility of specific genotype carriers to olanzapine-induced liver damage. Therefore, the aim of our study was to investigate if α-GST can be used for early detection of liver injury in patients receiving olanzapine and whether liver injury may vary between carriers of different *GST* and *OGG1* gene polymorphisms.

## PARTICIPANTS AND METHODS

The study population consisted of 30 patients aged 18–65 years with schizophrenia or schizoaffective disorders diagnosed at the Necmettin Erbakan University Psychiatry Clinic in Konya, Turkey according to the criteria of the Diagnostic and Statistical Manual of Mental Disorders IV (18).

Before the treatment started they were naïve to olanzapine. The treatment consisted of olanzapine alone over three months, and its use was verified with serum measurements. Excluded were the patients who had a concurrent psychiatric disorder, a history of drug dependence or mental retardation, and who were taking other potentially hepatotoxic substances. Table 1 shows their demographic particulars.

**Table 1:** Demographic and clinical characteristics of studied population (N=30)

	**Mean±SD / N (%)**
**Age/Age range** (years)	36.7±14.1/19–63
**Gender**	
Men	8 (26.7 %)
Women	22 (73.3 %)
**Baseline body weight** (kg)	64.8±12.3
**Height** (cm)	165.1±8.0
**Baseline body mass index** (kg/m^2^)	23.9±4.6
**Smoking**	10 (33.3 %)

The study was approved by the Ethics Committee of the Keçiören Education and Research Hospital (approval No. B.10.4. ISM.4.06.68.49), and written consent was obtained for all participants before admission to the study.

### Blood sampling

For genotyping and enzyme determination we collected venous blood (9 mL) in EDTA tubes for biochemical and DNA analyses before olanzapine treatment (T1, baseline), 10±3 days after starting olanzapine treatment (T2), and 3±1 months after treatment (T3), based on previously published studies (19, 20).

### Genotyping

DNA was isolated from the whole blood using the sodium perchlorate/chloroform method described in detail earlier (21). For *GSTM1* and *GSTT1* genotyping we used the multiplex polymerase chain reaction (PCR) and for *GSTP1* rs1695 *(313 A>G, Ile105Val)* and *OGG1* rs1052133 (*1245 C>G*, *Ser326Cys*) restriction fragment length polymorphism (RFLP)-PCR as described earlier (22).

### *GSTM1* gene polymorphisms

A partial gene deletion at the *GSTM1* locus on chromosome 1p13.3 (*GSTM1* null genotype) results in the complete absence of GSTM1 enzyme activity. To detect this deletion we used the following PCR primers: 5′-CGC CAT CTT GTG CTA CAT TGC CCG-3′ (primer 1), 5′-ATC TTC TCC TCT TCT GTC TC-3′ (primer 2), and 5′-TTC TGG ATT GTA GCA GAT CA-3′ (primer 3). PCR was performed in a total volume of 10 µL consisting of 100 ng of genomic DNA, 0.25 mmol/L dNTP, 1.5 mmol/L MgCl_2_, 0.4 µmol/L of each primer, and 1 unit of Taq DNA polymerase in 1 × PCR buffer. The PCR program consisted of a 2 min initial denaturation step at 94 °C, followed by 35 cycles of 1 min denaturation at 94 °C, 90 s annealing at 53 °C, and 2 min elongation at 72 °C. The final elongation was 10 min at 72 °C. The specific size of the *GSTM1* gene PCR product (231 bp) and a control band (158 bp) were assessed after electrophoresis on a 2 % agarose gel.

### GSTT1 gene polymorphism

The *GSTT1* gene deletion on chromosome 22q11.23 was determined with the PCR using the 5′-AGG CAG CAG TGG GGG AGG ACC-3′ forward primer and the 5′-CTC ACC GGA TCA TGG CCA GCA-3′ reverse primer. PCR was performed in a total volume of 10 µL consisting of 100 ng of genomic DNA, 0.2 mmol/L dNTP, 2 mmol/L MgCl_2_, 0.25 µmol/L of each primer, and 1 unit of Taq DNA polymerase in 1 × PCR buffer. The PCR program consisted of a 5 min initial denaturation step at 94 °C, followed by 30 cycles of 1 min denaturation at 94 °C, 1 min annealing at 60 °C, and 2 min elongation at 72 °C. The final elongation was 10 min at 72 °C. The specific sizes of the *GSTT1* product (138 bp) and a control band (158 bp) were assessed after electrophoresis on a 2 % agarose gel.

### GSTP1 gene polymorphism

An *A* to *G* polymorphism at a nucleotide in the *GSTP1* gene 313 results in an amino acid substitution (*Ile105Val*). This residue lies in the enzyme’s substrate-binding site, and the polymorphism has been shown to affect enzyme activity (22). To detect it we used the following primers: 5′-ACC CCA GGG CTC TAT GGG AA-3′ (forward) and 5′-TGA GGG CAC AAG AAG CCC CT-3′ (reverse). PCR was performed in a total volume of 20 µL consisting of 100 ng of genomic DNA, 0.2 mmol/L dNTP, 1.5 mmol/L MgCl_2_, 0.3 µmol/L of each primer, and 1 unit of Taq DNA polymerase in 1 × PCR buffer. The PCR program consisted of a 5 min initial denaturation step at 94 °C, followed by 30 cycles of 30 s denaturation at 94 °C, 30 s annealing at 57 °C, and 30 s elongation at 72 °C. The final elongation was 7 min at 72 °C. After the PCR, a 10 µL aliquot of the amplification product was digested overnight at 37 °C with 4 units of *BsmAI*. The digestion products were separated after electrophoresis on a 3 % NuSieve agarose gel, and the DNA fragments were visualised with 10 mg/mL ethidium bromide. The specific sizes of digestion products were as follows: *Ile/Ile* (wild) 176 bp, *Ile/Val* (heterozygous) 176, 93, and 83 bp, and *Val/Val* (mutant) 93 and 83 bp.

### OGG1 gene polymorphism

To genotype for the *OGG1 Ser326Cys* we used the following primers: 5′-ACT GTC ACT AGT CTC ACC AG-3′ (*OGG1*-forward 5′) and 5′-TGA ATT CGG AAG GTG CTT GGG GAA T-3′ (*OGG1*-reverse 3′). PCR was performed in a total volume of 20 µL consisting of 100 ng of genomic DNA, 0.25 mmol/L dNTP, 1.5 mmol/L MgCl_2_, 0.3 pmol of each primer, and 1 unit of Taq DNA polymerase in 1 × PCR buffer. The PCR program consisted of a 2 min initial denaturation step at 94 °C, followed by 33 cycles of 15 s denaturation at 94 °C, 30 s annealing at 60 °C, and 35 s elongation at 72 °C. The final elongation was 10 min at 72 °C. PCR yielded a 207 base pair product. After PCR, a 10 µL aliquot of the amplification product was digested overnight at 37 °C with 2 units *Fnu4HI*. The digestion products were separated after electrophoresis on a 3 % NuSieve agarose gel, and the DNA fragments were visualised with 10 mg/mL ethidium bromide. The specific sizes of digestion products were as follows: *Ser/Ser* (wild) 207 bp, *Ser/Cys* (heterozygous) 207, 107, and 100 bp, and *Cys/Cys* (mutant) 107 and 100 bp.

### Measurement of serum olanzapine concentrations

Serum olanzapine concentrations were measured with liquid chromatography–electrospray-ionisation-tandem mass spectrometry (LC-ESI-MS/MS) (Shimadzu LCMS-8030, Tokyo, Japan) following the validated method of Uřinovská et al. (23) with slight modifications as described earlier (24).

### Measurement of serum aminotransferases and α-GST levels

Serum levels of ALT and AST of patients were obtained from the hospital, whereas serum α-GST was measured with the enzyme-linked immunosorbent Assay (ELISA) according to the manufacturer’s instructions (Microplate Assay for GSTA Product Number: GS41, Oxford Biomedical Research Inc., Oxford, MI, USA).

### Statistical analysis

First we ran the power analysis with the G*power tool, release 3.1 (Heinrich Heine University, Düsseldorf, Germany) (25), which yielded a range from 0.85 to 0.93, confirming that our sample size was sufficient for further analysis. The obtained data were further analysed using the IBM SPSS Statistics version 17.0 (IBM Corporation, Armonk, NY, USA). Genotype distribution was checked against the Hardy-Weinberg equilibrium. The normality of distribution was determined with the Shapiro-Wilk test and the homogeneity of variances with the Levene test. Where applicable, descriptive statistics for continuous variables were expressed as means ± standard deviations (SD) or medians and quartile 1–3 range. The number of cases and percentages were used for categorical data. Mean differences between groups were compared using the Student’s *t*-test or Mann-Whitney *U* test. Differences between time points were evaluated for significance with the repeated measures analysis of variance (ANOVA) using the Wilks’ lambda or Friedman test where applicable. If they were statistically significant, we then ran the Bonferroni correction to control for type I errors for all possible multiple comparisons. Degrees of association between continuous variables were evaluated with Spearman’s rank correlation. A p-value of <0.05 was considered statistically significant.

## RESULTS

Table 2 shows mean olanzapine maintenance doses and the distribution of gene polymorphisms in the study population. We found no deviation from the Hardy-Weinberg equilibrium for any of the genotypes studied.

**Table 2:** Olanzapine maintenance doses and distribution of gene polymorphisms in the study population (N=30)

	**Mean±SD**
**Mean olanzapine maintenance dosing** (mg/kg)	0.13±0.052
**Mean serum olanzapine concentration (T2)**	0.42±0.565
**Gene polymorphisms**	**N (%)**
***GSTM1***	
Positive	12 (40.0 %)
Null	18 (60.0 %)
***GSTT1***	
Positive	22 (73.3 %)
Null	8 (26.7 %)
***GSTP1 Ile105Val***	
*Ile/Ile*	14 (46.7 %)
*Ile/Val + Val/Val*	16 (53.3 %)
***OGG1 Ser326Cys***	
*Ser/Ser*	16 (53.3 %)
*Ser/Cys+Cys/Cys*	14 (46.7 %)

### Liver enzyme levels

Table 3 shows that the first 10±3 days of treatment with olanzapine increased the levels of all enzymes compared to their baseline values (T2 vs T1). However, only ALT rose significantly at T2 compared to T1 and dropped to baseline levels at T3, whereas the other two enzymes did not change significantly.

**Table 3:** Liver enzyme levels in olanzapine-treated patients (N=30) measured at three time points

	**T1**	**T2**	**T3**	**p-value**
	**Median (Q1–3)**			
**α-GST (μg/L)**	2.1 (1.4–3.4)	3.4 (1.9–6.8)	2.8 (1.7–4.9)	0.239
**ALT (U/L)**	17.0 (11.0–20.2)^a^	25.0 (17.7–44.5)^a, b^	17.0 (13.7–24.0)^b^	**0.039**
**AST (U/L)**	19.5 (15.7–27.0)	25.0 (17.0–39.2)	19.0 (17.0–25.0)	0.692

T1 – before treatment (baseline); T2 – after 10±3 days of treatment; T3 – after 3±1 months of treatment.

a statistically significant difference between T1 and T2 (p=0.006; Friedman test).

b statistically significant difference between T2 and T3 (p=0.008; Friedman test)

Serum ALT was above the upper limit of normal (ULN of <40 U/L) in seven patients at T2 and in one patient at T3. Only one patient had ALT>2xULN at T2 (clinically mild elevation).

Serum AST levels were above ULN of <40 U/L in six patients at T2 and in one patient at T3. All elevations were below <2×ULN.

### Association between gene polymorphisms and liver enzyme levels

Tables 4–6 show no significant associations between gene polymorphisms and the levels of liver enzymes measured at the three time points, save for the association between the *GSTP1 Ile/Val + Val/Val* polymorphism and ALT (p=0.014).

**Table 4:** Association between gene polymorphisms and α-GST levels (μg/L) measured at the three time points in olanzapine-treated patients (N=30)

**Gene polymorphisms**	**T1**	**T2**	**T3**	p-value^*^
	**Median (Q1–3)**			
***GSTM1***				
Positive	1.7 (1.0–3.3)	2.8 (1.7–4.7)	2.2 (1.7–5.7)	0.717
Null	2.5 (1.4–3.5)	4.2 (2.1–7.2)	2.9 (2.0–4.3)	0.311
p-value^**^	0.368	0.415	0.692	
***GSTT1***				
Positive	2.0 (1.3–2.8)	2.9 (1.9–4.4)	2.9 (2.1–4.9)	0.186
Null	4.0 (1.4–9.8)	6.4 (2.1–10.1)	1.8 (1.4–6.5)	0.607
p-value^**^	0.118	0.156	0.420	
***GSTP1 Ile105Val***				
*Ile/Ile*	1.9 (1.3–2.8)	2.9 (1.8–6.2)	3.3 (1.7–6.5)	0.395
*Ile/Val+Val/Val*	2.6 (1.4–4.2)	4.0 (2.3–7.0)	2.4 (1.7–3.4)	0.305
p-value^**^	0.377	0.313	0.334	
***OGG1 Ser326Cys***				
*Ser/Ser*	2.8 (1.7–4.3)	3.6 (1.9–7.4)	2.9 (2.0–4.3)	0.646
*Ser/Cys+Cys/Cys*	1.6 (1.1–2.6)	3.4 (1.8–6.2)	2.3 (1.6–6.1)	0.257
p-value^**^	0.077	0.580	0.728	

T1 – before treatment (baseline); T2 – after 10±3 days of treatment; T3 – after 3±1 months of treatment.

* difference between time points within each gene polymorphism (significant if p<0.025; Friedman test, Bonferroni correction).

** difference between gene polymorphisms within each time point (significant if p<0.0167; Mann-Whitney *U* test, Bonferroni correction)

**Table 5:** Association between gene polymorphisms and ALT levels (U/L) measured at the three time points in olanzapine-treated patients (N=30)

**Gene polymorphisms**	**T1**	**T2**	**T3**	p-value^*^
	**Median (Q1–3)**			
***GSTM1***				
Positive	16.5 (12.2–18.5)	30.0 (15.7–43.2)	19.0 (10.7–25.7)	0.090
Null	17.5 (11.0–24.5)	23.5 (17.7–46.0)	16.0 (14.0–22.2)	0.183
p-value^**^	0.632	0.917	0.819	
***GSTT1***				
Positive	16.5 (11.0–19.0)	23.5 (16.7–34.2)	18.0 (14.7–23.2)	0.153
Null	17.5 (11.5–33.7)	35.0 (18.7–46.0)	14.5 (10.2–24.7)	0.223
p-value^**^	0.344	0.504	0.504	
***GSTP1 Ile105Val***				
*Ile/Ile*	17.0 (13.2–25.2)	21.0 (16.7–34.7)	17.0 (13.7–29.0)	0.323
*Ile/Val+Val/Val*	15.0 (11.0–19.0)	27.5 (19.0–45.5)^a^	18.0 (11.7–23.7)^a^	**0.014**
p-value^**^	0.608	0.355	0.822	
***OGG1 Ser326Cys***				
*Ser/Ser*	16.5 (11.2–18.7)	24.5 (17.2–38.5)	17.0 (14.2–20.0)	0.170
*Ser/Cys+Cys/Cys*	17.0 (10.7–26.7)	26.5 (17.2–51.0)	19.0 (12.2–29.5)	0.211
p-value^**^	0.918	0.697	0.697	

a statistically significant difference between T2 and T3 (p=0.003). T1 – before treatment (baseline); T2 – after 10±3 days of treatment; T3 – after 3±1 months of treatment.

* difference between time points within each gene polymorphism (significant if p<0.025; Friedman test, Bonferroni correction).

** difference between gene polymorphisms within each time point (significant if p<0.0167; Mann-Whitney *U* test, Bonferroni correction)

**Table 6:** Association between gene polymorphisms and AST levels (U/L) measured at three predetermined time points in olanzapine-treated patients (N=30)

**Gene polymorphisms**	**T1**	**T2**	**T3**	p-value^*^
	**Median (Q1–3)**			
***GSTM1***				
Positive	21.5 (16.5–29.2)	24.5 (16.0–39.7)	22.0 (16.2–32.0)	0.770
Null	19.0 (14.0–26.2)	25.5 (17.0–39.2)	19.0 (17.7–21.0)	0.765
p-value^**^	0.545	0.662	0.305	
***GSTT1***				
Positive	19.5 (15.7–24.5)	22.5 (16.7–39.2)	19.0 (16.7–25.0)	0.955
Null	23.0 (15.0–35.7)	33.0 (21.2–47.2)	20.5 (18.0–30.2)	0.368
p-value^**^	0.420	0.185	0.565	
***GSTP1 Ile105Val***				
*Ile/Ile*	19.0 (15.7–25.0)	24.5 (18.2–37.7)	20.5 (17.0–29.7)	0.982
*Ile/Val+Val/Val*	22.5 (15.0–27.0)	27.5 (17.0–39.7)	19.0 (16.5–21.7)	0.399
p-value^**^	0.608	0.580	0.334	
***OGG1 Ser326Cys***				
*Ser/Ser*	19.0 (15.7–26.2)	25.5 (17.5–39.7)	19.5 (18.0–21.7)	0.814
*Ser/Cys+Cys/Cys*	21.0 (15.5–30.2)	23.0 (16.5–39.2)	19.0 (16.0–30.7)	0.801
p-value^**^	0.667	0.580	0.984	

T1 – before treatment (baseline); T2 – after 10±3 days of treatment; T3 – after 3±1 months of treatment.

^*^ difference between time points within each gene polymorphism (significant if p<0.025; Friedman test, Bonferroni correction).

^**^ difference between gene polymorphisms within each time point (significant if p<0.0167; Mann-Whitney *U* test, Bonferroni correction)

## DISCUSSION

Previous studies have generally evaluated the importance of α-GST in individuals with acute and chronic liver disease (such as hepatitis, chronic liver disease and cirrhosis) and compared it with ALT and AST. It has been suggested that α-GST may be used to confirm ALT and AST results in hepatocellular damage, that is, as an indicator of advanced damage (26). A few case studies of olanzapine (27, 28) report patients developing olanzapine-induced elevation of liver enzymes and liver-related diseases, but the underlying mechanism was unknown. These enzymes returned to normal levels after the drug was discontinued.

Our findings do not confirm our hypothesis that olanzapine treatment significantly increases serum α-GST early into the treatment or that it can serve as an earlier biomarker of liver injury than ALT or AST in these patients. Instead, ALT turned out to be the only significant early biomarker of liver injury, however mild, which is in line with several earlier reports (20, 29).

Furthermore, we found no significant association between gene polymorphisms and liver enzyme levels, save for the one between the *GSTP1 Ile/Val + Val/Val* genotype and ALT, which suggests that carriers of this specific genotype might be at a higher risk of drug-induced liver injury. However, since our findings are limited to 30 patients, this and other associations between gene polymorphisms and liver enzyme levels call for further investigation in a much larger population sample.

Even so, our study points to the need to monitor all three enzymes in clinical practice to minimise the risk of liver injury in patients receiving long-term olanzapine treatment and is in line with a number of (case) reports of transient liver biochemistry abnormalities in olanzapine patients (27, 28, 30, 31, 32). More importantly, though, it calls for an investigation in a much larger sample to identify the real risks. Besides a small sample, our study is also limited in the sense that we have not considered other risk factors, such as obesity or age, since our study period was relatively short.

## CONCLUSIONS

Considering that the changes in ALT, AST, and α-GST levels were not significant, we cannot claim that olanzapine induced liver damage. However, the three biomarkers showed a similar rise and fall pattern, which suggests that α-GST could be used to monitor liver damage in olanzapine treatment along with ALT and AST. ALT turned out to be a superior biomarker to α-GST or AST, and the *GSTP1 Val/Val* genotype stood out as associated with higher ALT levels in olanzapine treatment. At this point, however, we cannot identify the underlying mechanism for this association. Perhaps a study of the kind in patients with liver disease causing severe changes in liver enzymes will provide the answer. In any case, further more comprehensive studies are needed to answer the questions raised by this one.
